# Post-treatment MRI aspects of photodynamic therapy for prostate cancer

**DOI:** 10.1007/s13244-014-0359-8

**Published:** 2014-10-07

**Authors:** M. Kulik, C. Nedelcu, F. Martin, S. Lebdai, M. C. Rousselet, A. R. Azzouzi, C. Aubé

**Affiliations:** 1 Department of Radiology, CHU Angers, 4 rue Larrey, 49100 Angers, France; 2 Department of Urology, CHU Angers, 4 rue Larrey, 49100 Angers, France; 3 Department of Anatomical Pathology, CHU Angers, 4 rue Larrey, 49100 Angers, France

**Keywords:** Photodynamic therapy, Prostate cancer, MRI

## Abstract

**Objectives:**

Photodynamic therapy is a new focal therapy for prostate cancer.

**Methods:**

In this technique, a photosensitising agent is introduced intravenously, then activated by local laser illumination to induce tumour necrosis. Treatment efficacy is assessed by magnetic resonance imaging (MRI).

**Results and Conclusions:**

We illustrate specific post-treatment MRI aspects at early and late follow-up with pathological correlations.

**Teaching points:**

• *Dynamic phototherapy is a new and promising focal therapy for prostate cancer.*

• *One-week MRI shows increased volume of the treated lobe and large, homogeneous necrosis area.*

• *Six-month MRI shows significant changes of the prostate shape and signal.*

• *Six-month MRI becomes “base line” appearance for further follow-up or monitoring.*

## Introduction

In 2012, with 417,000 new cases and 92,200 deaths, prostate cancer was the third most common cancer in Europe overall after female breast cancer and colorectal cancer [1]. In men only it was the most common cancer, followed by lung and colorectal cancers. The incidence of prostate cancer, especially for localised disease, has been growing since the 1990s, a dynamic that may be attributable to the widespread use of prostate-specific antigen (PSA) tests [2] and increasing life expectancies.

The management of prostate cancer is still under debate in the urological community. Radical prostatectomy, external beam radiotherapy or brachytherapy are still considered gold-standard treatments, but carry with them well-known side effects (urinary incontinence, erectile dysfunction, intestinal toxicity). Active surveillance and watchful waiting are attractive options for localised tumours [3]. Various focal treatments, such as phototherapy, cryotherapy, interstitial thermotherapy and dynamic high-frequency ultrasound (HIFU), are being evaluated [4, 5].

**Fig. 1:**
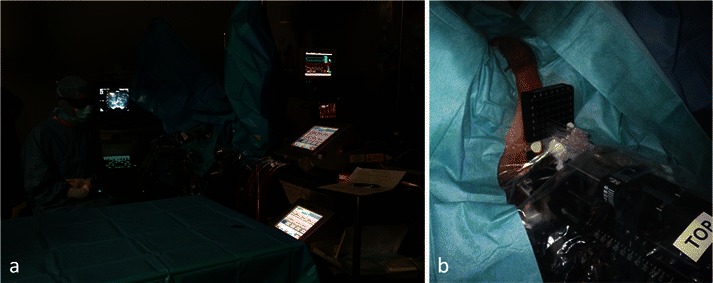
Procedure performed under general anaesthesia, in the darkness (**a**). Catheters were positioned into the prostate, using TRUS-image guidance in accordance with a previously devised MRI-based treatment plan. Cylindrically diffusing optical fibres were inserted into the catheters (**b**)

Photodynamic therapy is a recent technology wherein a photosensitising agent (WST11) is administered intravenously, then activated by light to induce tumour necrosis. The light is applied using laser optical fibres positioned within the prostate as determined by magnetic resonance imaging (MRI) planning. The procedure is performed under general anaesthesia, in the dark. Hollow, transparent, plastic brachytherapy catheters were positioned into the prostate, using TRUS-image guidance in accordance with a previously devised MRI-based treatment plan (Fig. 1). Cylindrically diffusing optical fibres were inserted into the catheters. Laser light was delivered to the prostate, using a multichannel diode laser. The single TOOKAD Soluble intravenous (i.v.) dose was a 10-min infusion, followed by continuous illumination of the prostate gland for between 20 and 25 min. The total duration of the whole procedure was ~2 h. The light activates the photosensitiser in the prostate, generating reactive oxygen species that activate thrombosis within the vessels. This results in obliteration of the microvessel anatomy with resultant deprivation of oxygen and nutrients to the tumour cells and surrounding prostate tissue in the treated area [6–8]. An optimal radius of 6.5 mm is expected around a 753-nm laser fibre [9]. The treatment appears promising for localised prostate cancer and benefits from a reasonably low rate of complications [10]. Currently, MRI is the reference imaging technique for the diagnosis of disease and follow-up after local treatment [11–13].

However, the changes induced by photodynamic therapy are poorly reported in the literature. Thus in the present pictorial review, we illustrate various MRI features in the follow-up of patients who underwent photodynamic therapy for localised prostate cancer. We studied early and late normal aspects, local complications and aspects of recurrence, with histopathological correlation.

## Iconography and imaging protocol

The iconography presented in this paper comes from a group of 77 patients enrolled by our institution in a prospective international multicentric study (phase II/III), and who received photodynamic therapy with WST11 (Tookad soluble) for localised prostate cancer.

Inclusion criteria are: men over 18 years old; diagnosed prostate cancer histologically proved on biopsies (systematic biopsies most of the time, rarely targeted biopsies); eligible for active surveillance; no prior treatment for prostate cancer; TNM stage up to cT2b- N0/Nx- M0/Mx; Gleason score ≤ 3 + 3, 3 + 4 accepted in certain conditions; PSA <10 ng/ml. The prostate cancer diagnosis was histological. All patients had a pre-treatment MRI then post-treatment MRIs at 1 week (early post-treatment) and 6 months (late post-treatment). Some of the patients also had a post-treatment MRI at 3 months. Systematic biopsies with TRUS (trans-rectal ultrasound) guidance were performed at 6 months to explore correlations to MRI features. In case of positive biopsy at 6 months, function of the Gleason score, different options were proposed to the patients: treatment of the other lobe, retreatment on the same lobe, active surveillance or surgery.

## MRI protocol

All MRIs were performed with a 1.5-T MRI system (GE Excite, General Electrics, Milwaukee, IL). An external body surface coil (eight channels) was used, with a field of view (FOV) equal to 24 cm, except for the dynamic contrast-enhanced sequences for which FOV was 42 cm.

The imaging protocol included the following sequences: axial and coronal FRFSE T2 with fat saturation (4-mm slice thickness), axial FSE T1 (4-mm slice thickness), axial diffusion-weighted (*b* = 600 s/mm^2^); a three-dimensional (3D) dynamic contrast-enhanced T1 sequence (LAVA) with fat saturation after intravenous injection of 20 ml gadoterate meglumine (Dotarem, Guerbet, France), followed by axial and sagittal fat saturation contrast-enhanced FSE T1-weighted images. These last sequences were systematically realised, to better define boundaries of necrosis, especially in case of extra prostatic necrosis.

Pre- and post-treatment MRI exams were analysed on an imaging work station (Fujifilm Synapse 3D). We analysed MRI modifications for the areas treated with photodynamic therapy (optical fibre disposition map) and the histopathological results of systematic 6-month biopsies. Some patients underwent prostatectomy after the phototherapy treatment, permitting a more accurate radiological-pathological correlation.

PIRADS classification was not used for treatment planning. Pre-treatment MRI served only to plan the anatomic distribution of the optic fibres into the lobe to be treated, targeting the zones of positive biopsy. The PIRADS criteria were used for the follow-up MRI analyses.

### Pre-treatment MRI

Pre-treatment MRI was performed as a planning procedure to determine the position and the number of optical fibres and to exclude any loco-regional extension that would have contraindicated the treatment.

**Fig. 2:**
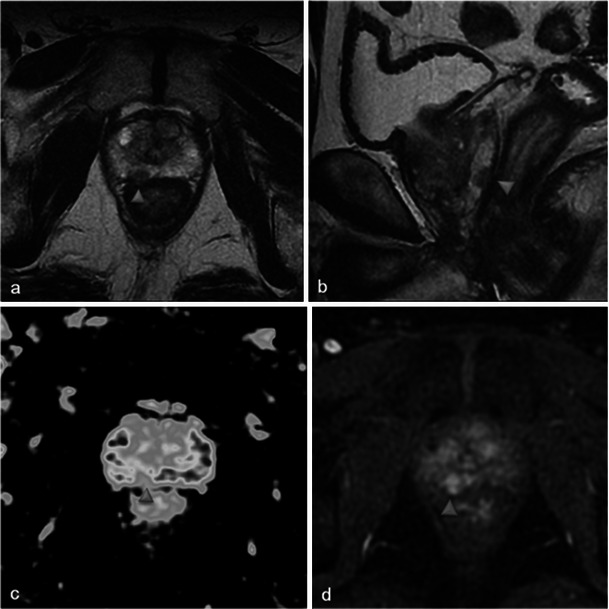
Pre-treatment MRI of a 65-year-old patient with a PSA level of 9.7 ng/ml and a positive biopsy in the right prostate apex (adenocarcinoma Gleason 6). Axial and sagittal T2-weighted images (**a**, **b**) show a hypointense nodule in the right peripheral prostate with restriction on the apparent diffusion coefficient map of the diffusion-weighted image (**c**, low signal intensity) and early enhancement on the axial dynamic contrast-enhanced MR image (**d**, *arrowhead*)

Only small tumours with low aggressiveness (Gleason 6) were considered for photodynamic therapy and thus not all of them were visible. When tumours were visible, the classical aspect was a hypointense nodule in T2-weighted MR images, with restriction on the apparent diffusion coefficient map and early enhancement on dynamic contrast-enhanced MR images (Fig. 2).

### Early post-treatment MRI features

**Fig. 3:**
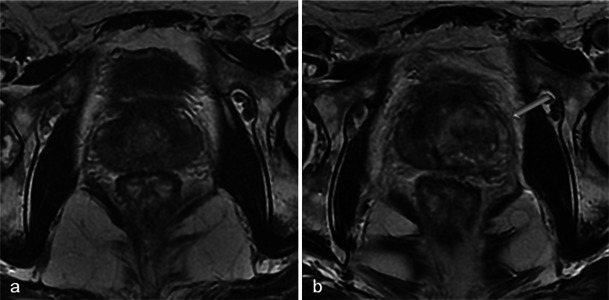
Oedema and necrosis. Left lobe photodynamic therapy in a 65-year-old patient with a PSA level of 6.2 ng/ml and two positive biopsies in the left lobe—Gleason 6 (3 + 3) adenocarcinoma. **a** Pre-treatment axial T2-weighted image shows diffuse bilateral hyposignal of the peripheral prostate without focal nodularity. **b** Day-7 post-treatment axial T2-weighted image shows a heterogeneous signal of the treated lobe extending to the peripheral and transitional zone (*arrowhead*). Note that the signal of the right lobe peripheral zone is unchanged compared with the pre-treatment image

One-week MRI examinations show T2 heterogeneous signals in the treated lobes (Fig. 3) that are related to the oedema and ischaemic modifications induced by phototherapy. These modifications are secondary to local intravascular coagulation triggered by the photosensitising agent when exposed to laser light.

**Fig. 4:**
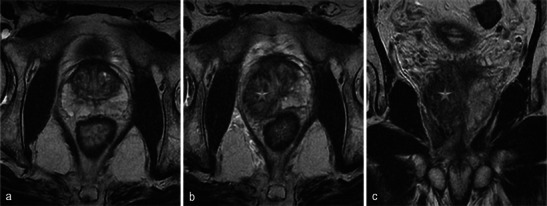
Volume increase of the treated lobe. A 65-year-old patient with a middle right lobe positive biopsy. Pre-treatment axial T2 sequence (**a**) shows a normal prostate. Axial (**b**) and coronal (**c**) day-7 post-treatment T2-weighted images show an asymmetrical increase of the volume of the treated lobe (*star*)

MRI sequences show an increased volume of the treated lobe. When the procedure is unilateral, the volume variation is obvious and particularly well visualised in T2-weighted sequences (Fig. 4) because of their clear delineation of prostate borders.

**Fig. 5:**
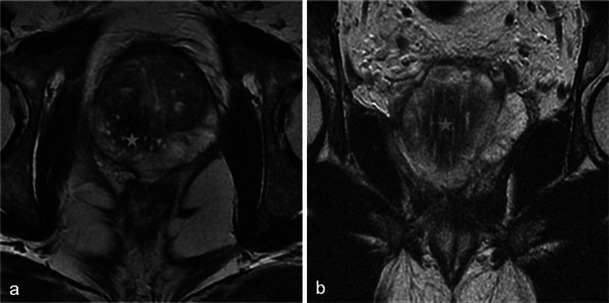
Path of optical fibres. Right prostate lobe treatment for adenocarcinoma Gleason 6 (3 + 3) in a 62-year-old patient with a PSA level of 9 ng/ml. Tumour was not visible on pre-treatment MRI. The paths of the optical fibres in the treated area are visible on the day-7 post-treatment axial T2-weighted image (**a**) as small hyperintense spots corresponding to thin linear bands (*star*) in the coronal view (**b**)

Among all heterogeneities within the treated lobe, besides the loss of physiological distinction between transitional and peripheral prostate tissue in T2 images, the paths of the optical fibres can be observed as small hyperintense spots in the axial plane and linear bands in the sagittal and coronal planes (Fig. 5).

**Fig. 6:**
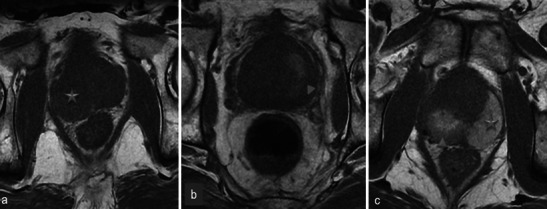
Haemorrhage within treated lobes is variable. **a** Day-7 post-treatment axial T1 MR image in a 64-year-old patient with right lobe photodynamic therapy shows an asymmetric prostate (*star*) with increased volume of the treated lobe but without any spontaneous high signal intensity evoking haemorrhage in the treated area. **b** A 72-year-old patient with left lobe treatment. Day-7 post-treatment axial T1 shows a hyperintense area (*arrowhead*) corresponding to post-treatment haemorrhagic suffusions. **c** Right lobe phototherapy in a 59-year-old patient. Day-7 post-treatment axial T1-weighted MR image shows a spontaneous high-signal-intensity area in the right lobe (*arrowhead*), but also an extended spontaneous high signal intensity in the left (untreated) lobe (*star*)

On T1-weighted images before gadolinium injection, hyperintense, poorly defined areas can be identified. These areas are related to haemorrhagic suffusions (Fig. 6) and make T1-weighted contrast-enhanced images difficult to analyse; in this situation the use of image subtraction may be helpful.

**Fig. 7:**
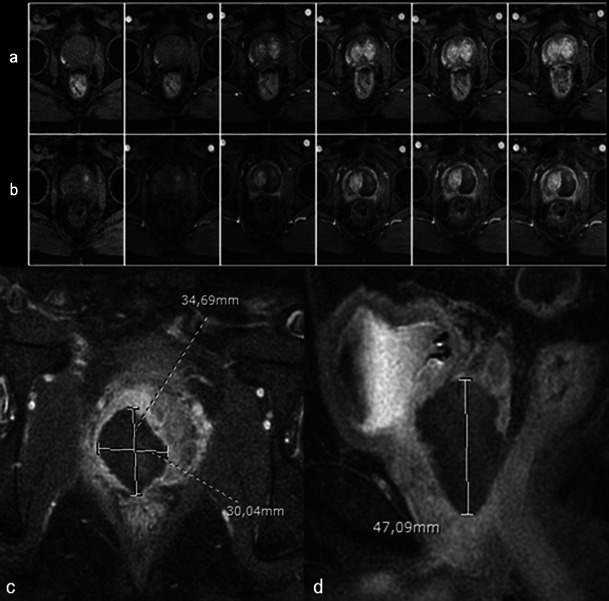
Well-defined necrosis boundaries. A 72-year-old patient with a PSA level of 4.5 ng/ml treated for Gleason 6 (3 + 3) left lobe adenocarcinoma. Axial dynamic contrast-enhanced images before treatment (**a**) show a zone of post-biopsy haemorrhage on the right lobe without any suspicious enhancement. Day-7 post-treatment left MRI (**b**) shows no enhancement of the left treated lobe (necrosis). Note the extended periprostatic enhancement that surrounds the whole gland and indicates inflammation. Right lobe treatment for Gleason 6 adenocarcinoma: day-7 post-treatment axial (**c**) and sagittal (**d**) FS contrast-enhanced images show a large homogenous hypointense non-enhanced area in the treated lobe (necrosis) with well-defined and highly enhanced boundaries

**Fig. 8:**
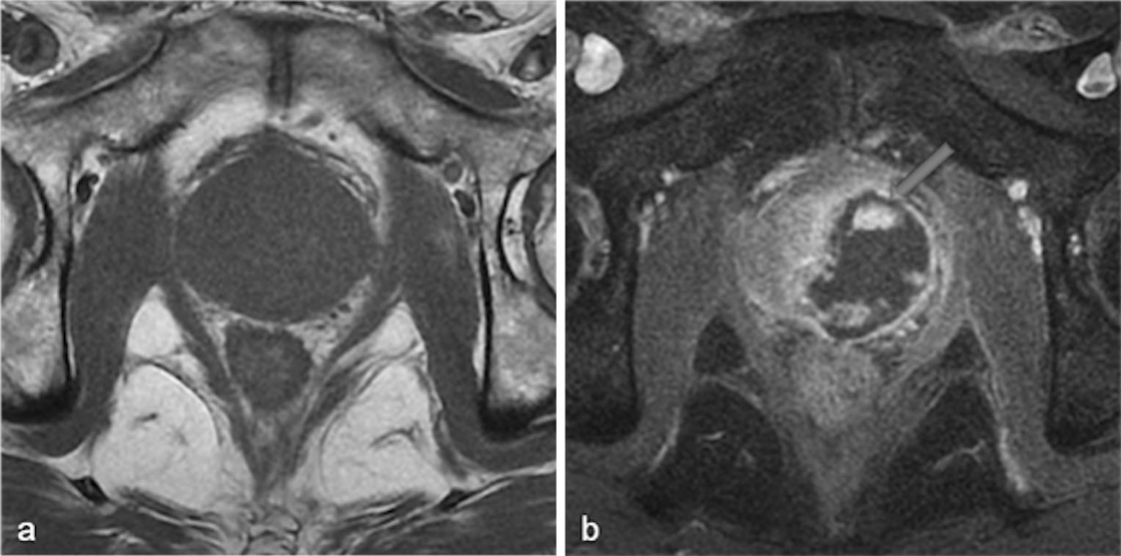
Focal spared tissue. Left lobe photodynamic therapy in a 66-year-old patient. Axial T1 (**a**) and contrast-enhanced axial T1 FS (**b**) images show islands of spared tissue (*arrowhead*) that enhance within the treated area

Necrosis is best defined on T1-weighted FAT-SAT contrast-enhanced images. It appears as non-enhancing areas [14] with irregular but well-defined and highly enhanced edges (Fig. 7). In most cases, a treated lobe presents a single, large, homogeneous necrosis area on T1 contrast-enhanced sequences. However, in some cases, islands of spared tissue may be observed with high early enhancement (Fig. 8). This aspect should not be wrongly analysed as residual tumour [14].

**Fig. 9:**
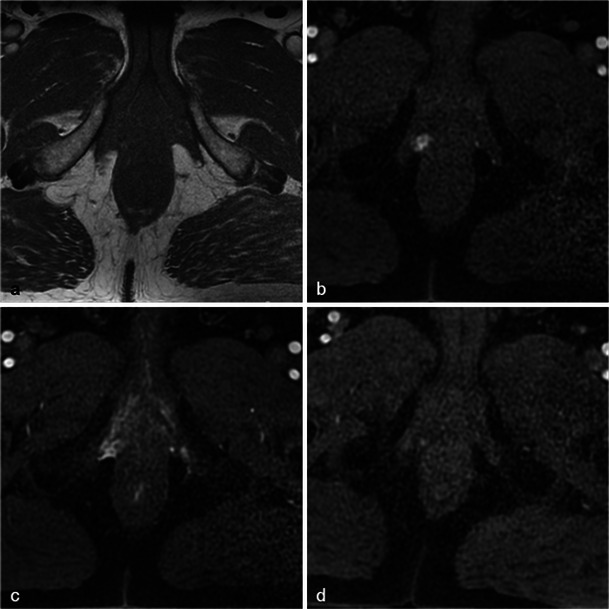
Suspected residual tumour. Day-7 post-treatment dynamic contrast-enhanced fat-saturated axial T1-weighted MR image showing early nodular enhancement in the prostate apex suggestive of residual tumour away from the treated area. Diffusion-weighted MRI was not contributive. The nodule was no longer visible on the 3-month post-treatment and 6-month post-treatment MRIs, and the 6-month post-treatment biopsy was negative

Another pitfall to be avoided is focal inflammatory enhancements in the treated lobe presenting a nodular feature (Fig. 9). These lesions are usually no longer present on 3-month post-treatment MRIs.

**Fig. 10:**
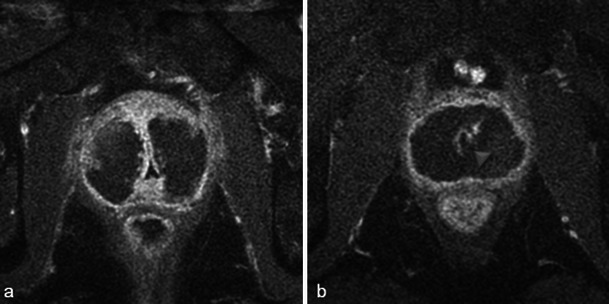
Periurethral enhancement ring. Day-7 post-treatment axial T1 FS contrast-enhanced MR images for a bilaterally treated patient show necrosis in both lobes. The intraprostatic urethra presents a continuous enhancement ring (**a**). A rupture of this ring enhancement (*arrowhead*) after photodynamic therapy is considered suspicious of urethral wall necrosis (**b**). No urinary complications were identified in this patient during close clinical follow-up

In cases with concomitant bilateral treatment, the 1-week MRI shows the same types of modifications but they are symmetrical. A particularity of bilateral treatment is the higher risk of urethral wall necrosis. Normally, the intraprostatic urethra exhibits a continuous ring of peripheral enhancement [14]. A discontinuous ring (Fig. 10) on the 1-week post-photodynamic therapy MRI is thus suggestive of urethral wall necrosis. In this situation, despite earlier animal studies reporting structural and functional resistance of the urethra to photodynamic therapy [15], close clinical monitoring is essential.

Finally, it is important to note that on the 1-week post-therapy images the surroundings of the gland may also show enhancement due to local inflammation.

### Late post-treatment MRI features

**Fig. 11:**
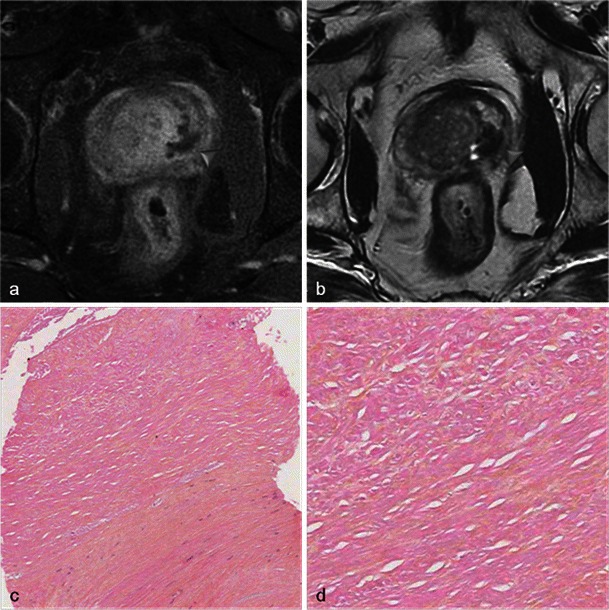
Residual necrosis. Six-month post-treatment MRI in a patient treated by left photodynamic therapy. Axial T1 FS contrast-enhanced (**a**) and axial T2-weighted images (**b**) show persistence of a low signal intensity area with no enhancement (*arrowhead*) corresponding to a small area of residual necrosis (coagulation necrosis). Pathology slides from biopsy at low (**c**) and high magnification (**d**) show loose connective tissue coreless, with cell ghosts, characteristic of coagulation necrosis

At 6 months after photodynamic therapy, important changes of the prostate shape and signal are found. Small areas of residual necrosis may still be present in the treated lobe, corresponding to coagulation necrosis (Fig. 11).

**Fig. 12:**
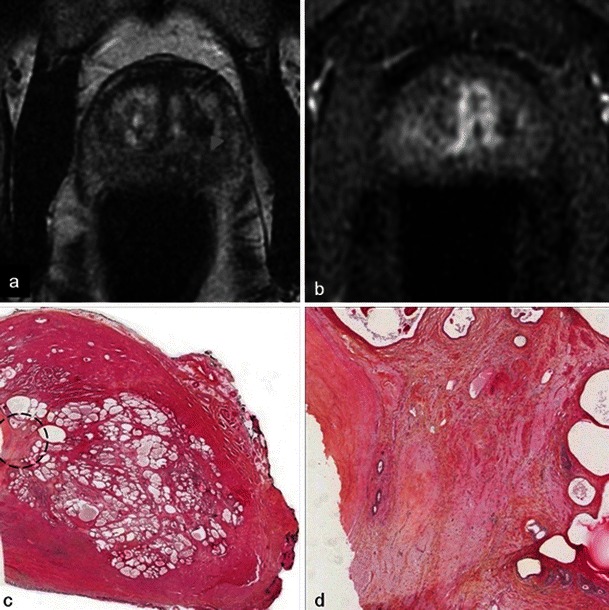
Fibrous scar. Axial T2-weighted MR (**a**) and axial T1 FS contrast-enhanced (**b**) images, in a case of bilateral photodynamic therapy show a post-treatment pattern, with a small area of low signal intensity, without enhancement, corresponding to fibrous scar tissue (*arrowhead*). Low power (**c**, **d**) images from radical prostatectomy show a focus of sclerotic and hyaline necrosis on the left lobe suggesting a 7 × 5-mm therapy scar

Classically, the volume of the treated lobe decreases, sometimes to less than the volume of the lobe before treatment. This is due to the apparition of retractile fibrous scar tissue (Fig. 12).

**Fig. 13:**
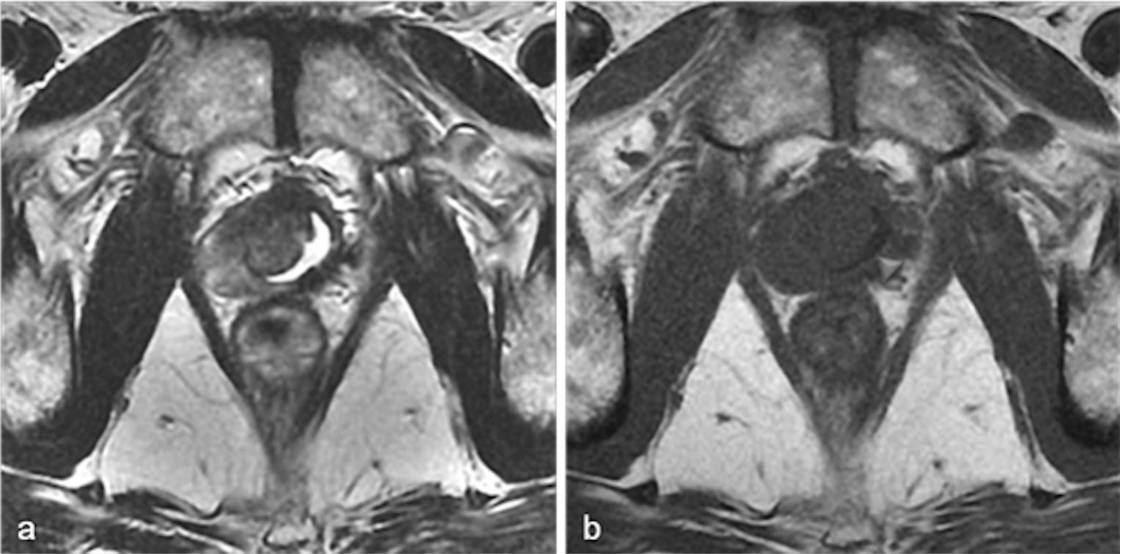
Residual fluid cavity. Six-month post-treatment follow-up MRI in a patient with left photodynamic therapy. Axial T2-weighted (**a**) and T1-weighted (**b**) MR images show an irregularly shaped residual fluid cavity in the treated lobe, with a low signal on T1 (*arrowhead*) and a high signal on T2

Residual, irregularly shaped fluid cavities related to atrophy in the treated lobe are frequently seen (Fig. 13). On T2-weighted images, the normal hypersignal of the peripheral prostate is replaced by the irregular hyposignal of the scar.

**Fig. 14:**
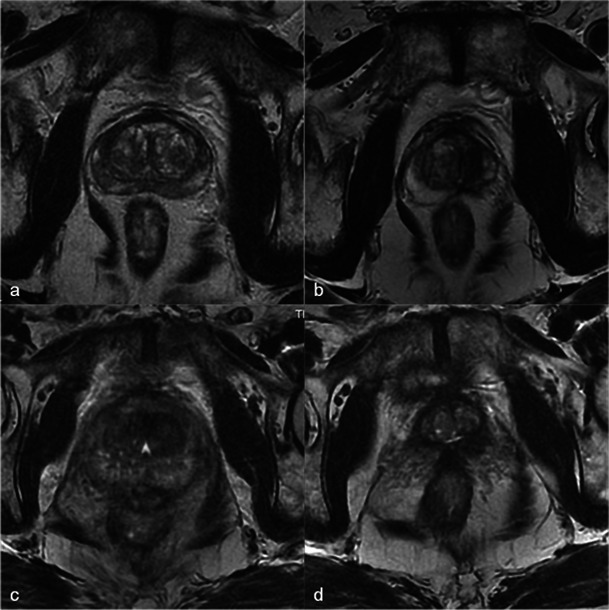
Asymmetry/symmetry. Axial T2-weighted MR images from before treatment (**a**) and 6 months after treatment (**b**) in a 65-year-old patient with left unilateral photodynamic therapy illustrate post-treatment asymmetric prostate due to unilateral scar tissue. However, pre-treatment (**c**) and 6-month post-treatment (**d**) MRIs in a case of bilateral treatment (71-year-old patient) illustrate a symmetrical aspect. Note the disappearance of the peripheral zone of the treated lobes on the 6-month MRIs

Unilateral treatment induces an asymmetric prostate, whereas bilateral treatment results in a small, heterogeneous, but symmetrical prostate (Fig. 14).

**Fig. 15:**
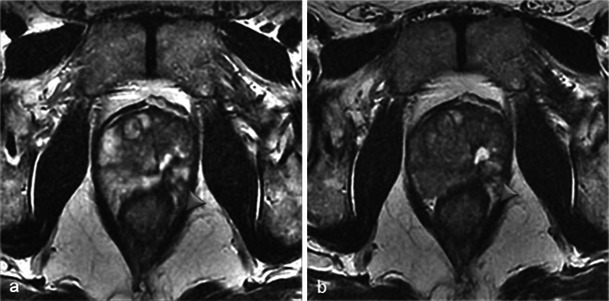
Unchanged scar appearance over time. Six-month post-treatment axial T2 images in a 66-year-old left photodynamic therapy patient show left lobe atrophy (particularly for the peripheral prostate) as well as a small fluid cavity (*arrowhead*) in the middle of the scar. There was no change in this aspect at 1 year (**b**)

**Fig. 16:**
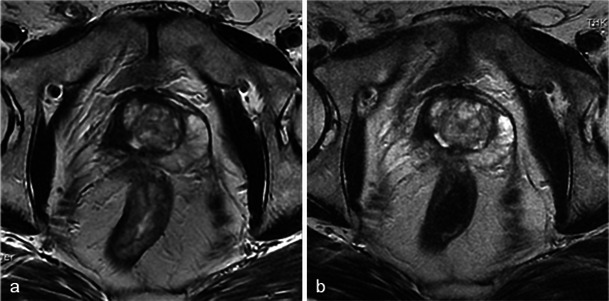
Unchanged scar appearance over time. Six-month (**a**) and 3-year (**b**) post-treatment MRI for right photodynamic therapy. The 6-month and 3 year axial T2-weighted images show the same aspect: atrophy of the right lobe with attraction of the urethra, and the disappearance of normal peripheral prostate hyperintensity. Treatment resulted in a lesser modification of the transitional zone compared with the contralateral lobe

Furthermore, the aspect observed at 6 months is definitive (Figs. 15 and 16). We thus underline that the 6-month MRI is very important as it will become the new “base line” appearance for any further follow-up or monitoring.

**Fig. 17:**
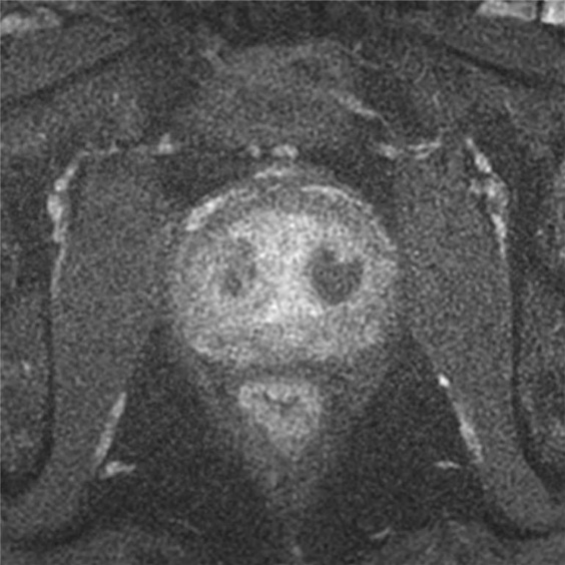
Three-month post-treatment MRI in a 69-year-old patient with concomitant bilateral treatment for prostate adenocarcinoma, Gleason 6 (PSA level of 8.14 ng/ml, 1/12 positive biopsy at right apex). Axial T1 FS contrast-enhanced image (**a**) shows the persistence of two small areas of residual necrosis (*arrowhead*)

Three-month MRIs are not pertinent for treatment follow-up as they tend to show aspects that are between early necrosis and late fibrosis and thus difficult to analyse (Fig. 17).

### Post-retreatment features

**Fig. 18:**
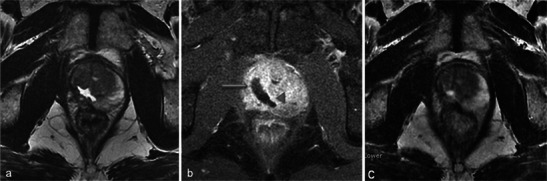
Retreatment of the same lobe. A 60-year-old patient with retreatment of the right lobe. Day-7 post-retreatment axial T2-weighted MR image (**a**) and axial T1 FS contrast-enhanced MR image (**b**) show a slight volume increase of the treated lobe compared with the first procedure; the area of prostate necrosis presents two different intensity levels: the residual fluid cavity (*arrowhead*) and tissue necrosis (*arrow*), with less low intensity. Note, 6 months post first treatment axial T2-weighted MR image (**c**) corresponding to pre-retreatment aspect

The indication criteria of retreatment are positive biopsies in treated or contralateral lobe during the follow-up, with Gleason score ≤ 3 + 3 or 3 + 4 in certain conditions. When ipsilateral retreatment is needed, the modifications seen after 1 week are similar to those seen 1 week after the initial treatment (Fig. 18). After 6 months, the atrophy in the retreated lobe is more marked than after the first treatment.

**Fig. 19:**
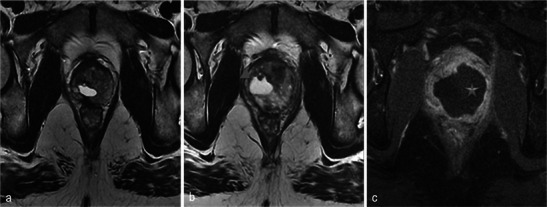
Contralateral treatment. A 56-year-old patient, with initial right photodynamic therapy, then left therapy in a second intervention. Pre-left therapy (**a**) and day-7 post-left therapy (**b**) axial T2-weighted images; day-7 post-left therapy (**c**) axial T1 FS contrast-enhanced MR image. On the left lobe, a heterogeneous T2 area with low signal in T1-weighted image, unenhanced, corresponding to the recent tissue necrosis (*star*). In the right lobe the T2 hyperintense old cystic cavity increased in size (*arrowhead*)

When a renewed intervention is for the contralateral lobe (Fig. 19) the asymmetric aspect is more pronounced at 1 week because of the atrophy of the previously treated lobe, but at 6 months, this asymmetry disappears and the prostate takes on the same appearance as a concomitant bilateral treatment.

### Complications

Photodynamic therapy appears to have a reasonably low rate of complications [10]. In a study on different types of focal therapies performed in a cohort of men with low-risk prostate cancer, the overall complication rate was 13 %, with only two Clavien-Dindo grade 3 complications. (Clavien Dindo is a grading system from 1 to 5, used for the classification of surgical complications; grade 3 or more are serious complications: grade 3—complication which requires surgical, endoscopic or radiological intervention—up to grade 5, which corresponds to the death of the patient). In that series, there were no complications described for the patients who received photodynamic therapy [16]. Azzouzi et al. [8] found that most treatment-emergent adverse events (TEAEs) post photodynamic therapy were mild or moderate and only 9 % of patients reported serious TEAEs (Table 1).

**Table 1:** Treatment-emergent adverse events (TEAEs) of photodynamic therapy. Data from Azzouzi et al. study [8]

Mild TEAE	Serious TEAE
Dysuria	Prostatitis
UTI (urinary tractus infection)	Haematuria
Urinary retention	Epididymo-orchitis
Constipation	Cystoprostatitis
Perineal pain	Ischaemic optic neuropathy
	Inflammatory prostatic cyst

The main post-photodynamic therapy complication is extraprostatic necrosis, defined as a lack of enhancement on T1-weighted contrast-enhanced MR images [14] involving the different structures surrounding the prostate.

**Fig. 20:**
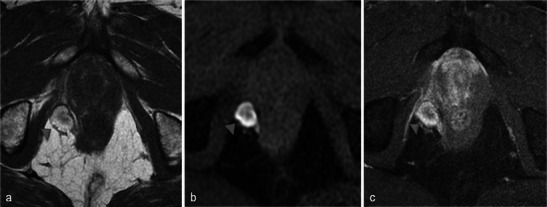
Periprostatic fatty tissue. A 62-year-old patient who had underwent left photodynamic therapy 1 year earlier, and right treatment 7 days earlier. Axial T1-weighted (**a**), axial T1 FS unenhanced (**b**) and contrast-enhanced (**c**) images show a nodular lesion (*arrowhead*) located in the right rectoprostatic angle, with a high intensity T1 FS signal, unenhanced, corresponding to focal haemorrhagic necrosis of periprostatic fatty tissue

**Fig. 21:**
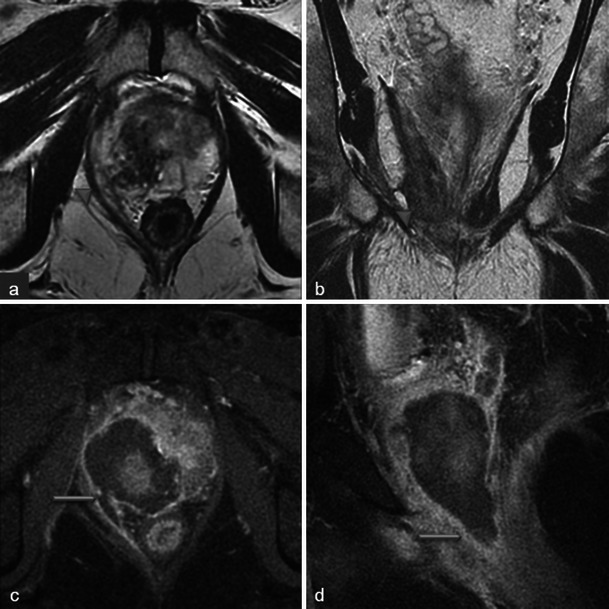
Levator ani muscle. Right photodynamic therapy in a 65-year-old patient for Gleason 6 adenocarcinoma (two positive biopsies in right prostate). Day-7 post-treatment axial (**a**) and coronal (**b**) T2-weighted MR images visualise a heterogeneous high signal of the right puborectalis muscle (*arrowheads*); axial and sagittal T1 FS contrast-enhanced images (**c**, **d**) show a low-signal well-defined area surrounded by a hyperintense enhanced border after gadolinium injection (*arrows*) corresponding to extraprostatic necrosis affecting the right levator ani muscle

**Fig. 22:**
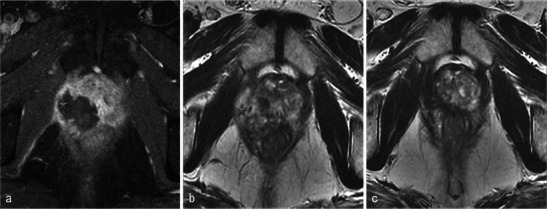
Levator ani muscle. Day-7 post-treatment axial T1 FS contrast-enhanced (**a**) and axial T2-weighted (**b**) MR images after right photodynamic therapy show extension of necrosis to puborectalis muscle (*star*); at the 6-month post-treatment MRI follow-up, the axial T2 image (**c**) illustrated the complete disappearance of the area of necrosis (*arrowhead*). No anal functional abnormalities were seen in this patient

When present, necrosis most frequently extends to periprostatic fatty tissue (Fig. 20). The muscles close to the prostate can also be affected, especially the levator ani (Fig. 21). However, in our experience with this situation, anal functional abnormalities are not observed and a normal aspect is recovered after 6 months (Fig. 22).

**Fig. 23:**
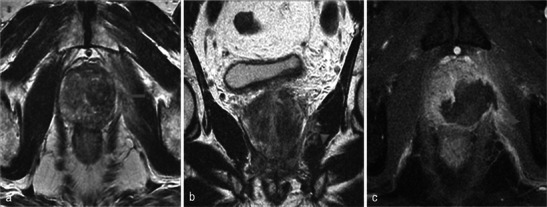
Internal obturator muscle. Left photodynamic therapy in a 66-year-old patient with Gleason 6 adenocarcinoma (pre-treatment/day-7 post-treatment/6-month post-treatment). Axial (**a**) and coronal (**b**) T2-weighted MR images show high signals of the left internal obturator muscle (*arrowhead*) and the puborectalis muscle (*arrow*). Extraprostatic necrosis extending to muscles is well defined on the T1 FS contrast-enhanced image (**c**, flash)

**Fig. 24:**
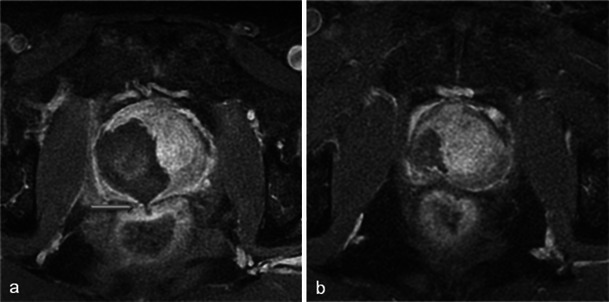
Anterior rectal wall. Right focal treatment for adenocarcinoma in a 65-year-old patient. Day-7 post-treatment (**a**) and 6-month post-treatment (**b**) axial T1 FS contrast-enhanced MR images show the presence of a thin line of necrosis extending to the anterior rectal wall (*arrow*), limited to the muscularis propria. Note the complete recovery of the rectal wall at 6 months. Also no fistulae were clinically identified

Extraprostatic necrosis may also affect the internal obturator muscle (Fig. 23) and rarely it may extend to the anterior rectal wall (Fig. 24). In our experience, fistulisation does not seem to occur and the rectal wall recovers its normal aspect on the 6-month follow-up MRI.

**Fig. 25:**
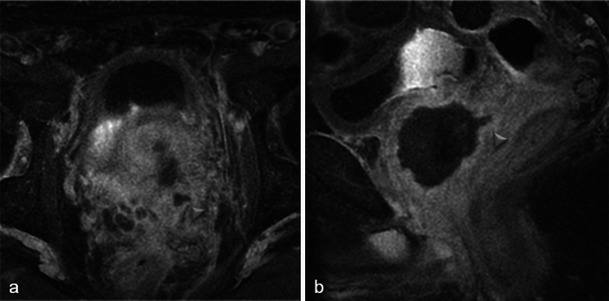
Seminal vesicle. Left photodynamic therapy in a 56-year-old patient. Day-7 post-treatment axial (**a**) and sagittal (**b**) T1 FS contrast-enhanced MR images show a small extension of necrosis to the left seminal vesicle (*arrowhead*) by extraprostatic sliding of an optical fibre

Sliding of the optical fibre may result in an extension to a seminal vesicle (Fig. 25).

**Fig. 26:**
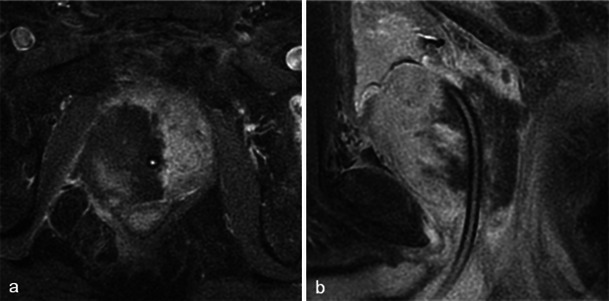
Urethral necrosis. Right photodynamic therapy in a 60-year-old patient with a PSA level of 12.9 ng/ml and Gleason 6 adenocarcinoma (positive base biopsies, right middle prostate). Day-7 post-treatment axial (**a**) and sagittal (**b**) T1 FS contrast-enhanced MR images do not show the normal periurethral ring of enhancement; the patient developed acute urinary retention treated by urinary catheter

As mentioned earlier, necrosis may affect the intraprostatic urethra; a break of the periurethral enhancement ring or its complete disappearance at 1 week is an interesting sign suggestive of urethral parietal necrosis (Fig. 26).

### Recurrence in the treated area

MRI detection of tumour recurrence in the treated area is made difficult by the loss of the normal hyperintensity of the peripheral prostate in T2-weighted images and the signal of the scar.

Thus, after photodynamic therapy, the diagnosis of recurrence is mainly based on PSA levels and systematic biopsy.

**Fig. 27:**
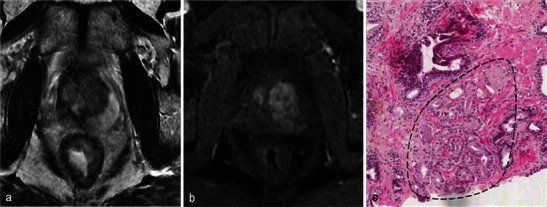
Small recurrence not seen on MRI. Patient with a positive right base prostate biopsy (3-mm focus). Six-month post-treatment axial T2-weighted (**a**) and axial dynamic contrast-enhanced (**b**) images show atrophy of the right lobe including the peripheral prostate; no suspicious nodules are visible. Pathology slide at high magnification (**c**) of a right base prostate biopsy, shows a small glandular focus (*circled*) between normal glands, lined by a single layer of cells

**Fig. 28:**
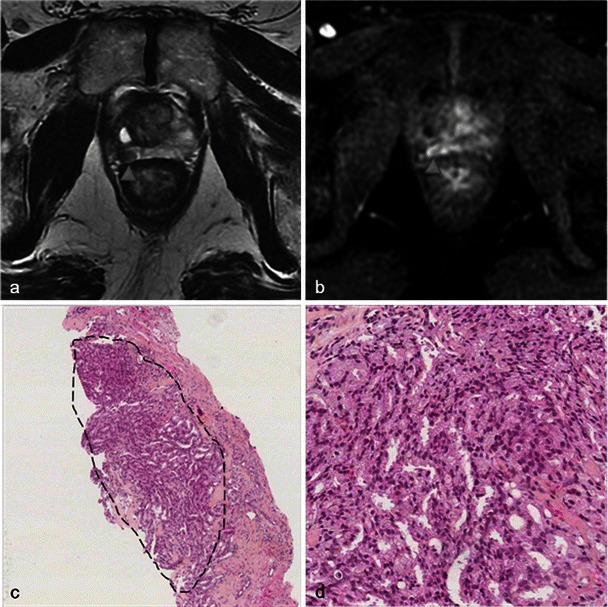
Recurrence seen on MRI. Right photodynamic therapy in a patient with Gleason 6 adenocarcinoma. Six-month post-treatment axial T2 (**a**) image shows a hypointense nodule of the right peripheral prostate and the axial dynamic contrast-enhanced (**b**) image illustrates an early enhancement after gadolinium injection of the nodule suggestive of recurrence (*arrowhead*). Low-power (**c**) and high-power (**d**) pathology slides of a right base prostate biopsy show small irregular glands lined by a single layer of cells, confirming the presence of an infiltrating Gleason 7 adenocarcinoma with perineural invasion

**Fig. 29:**
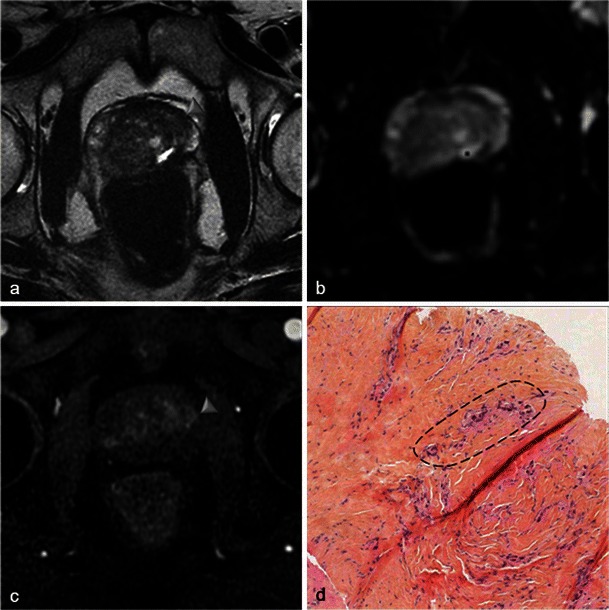
Mismatch. PSA increase in a patient treated by left photodynamic therapy 1 year earlier. MRI was performed: axial T2 (**a**), axial ADC (**b**) and axial T1 dynamic contrast-enhanced (**c**) MR images show a suspicious left median focus with T2 hyposignal, early enhancement and mild diffusion restriction on ADC map (*arrowhead*). The location of suspicious lesion seen on MRI is indicated on a prostate map. Targeted biopsy was performed with TRUS. Pathology slide at high magnification (**d**) shows a 1-mm tumour focus in a scar tissue. Crushed appearance of several glands (*circled*), suspect, confirmed by immunohistochemical study, and surrounded by thick collagen fibres (fibrous scar). The nodule on MRI corresponds to the scar tissue; the tumour focus is too small to be seen on MRI

There is no reliable feature of recurrence and cases of small unaggressive recurrence are usually not visible on MRI (Fig. 27). Early dynamic contrast-enhanced images and apparent diffusion coefficient mapping may be useful for detecting recurrence (Fig. 28) but they lack reliability. Therefore, any suspicious nodule seen on follow-up MRI should be biopsied (Fig. 29). Targeted biopsies with TRUS are performed in nodules presenting PIRADS criteria on follow-up MRI.

## Conclusions

Photodynamic therapy is a new and promising focal treatment for localised prostate cancer. Early and late post-treatment MRI aspects are specific. Necrosis is obtained at 1 week and the final post treatment aspect at 6 months. Knowledge of these features permits the appreciation of treatment efficacy and the accurate diagnosis of complications or recurrence.
